# Equilibrium folding dynamics of *me*ACP in water, heavy water, and low concentration of urea

**DOI:** 10.1038/s41598-017-16449-4

**Published:** 2017-11-23

**Authors:** Yang Zhou, Daiwen Yang

**Affiliations:** Department of Biological Sciences, National University of Singapore, 14 Science Drive 4, Singapore, 117543 Singapore

## Abstract

Many proteins fold in apparent two-state behavior, as partially folded intermediates only transiently accumulate and easily escape detection. Besides a native form and a mainly unfolded form, we captured a partially unfolded form of an acyl carrier protein from *Micromonospora echinospora* (*me*ACP) in the folding/unfolding equilibrium using chemical exchange saturation transfer NMR experiments. The C-terminal region of the partially unfolded form is mainly folded and the N-terminal is unfolded. Furthermore, to understand how the folding process of *me*ACP is influenced by solvent environments, we compared the folding dynamics of *me*ACP in D_2_O, H_2_O and low concentration of urea. As the environment becomes more denaturing from D_2_O to H_2_O and then to urea, the unfolded state becomes increasingly populated, and the folding rate decreases. Adding a small amount of urea, which does not change solvent viscosity, has little effects on the unfolding rates, while changing H_2_O to D_2_O reduces the unfolding rates possibly due to the increase of solvent viscosity. The quantified solvent effects on the protein folding Gibbs energy and activation energy suggest that the transition state of folding may have a similar structure to the native state of the protein.

## Introduction

Protein molecules exist in multiple conformational states under native conditions. The dominant folded state with the lowest free energy is in dynamic equilibrium with minor states such as unfolded state and partially unfolded intermediate states^1–6^. Studying the conformational exchanges among different states of a protein can reveal structure-function relationship^6–8^ or/and help to understand protein folding^3,4,9^.

Protein folding to biologically functional conformations is important to all living organisms. Misfolding may lead to fatal diseases. Our understanding of protein folding has advanced in the past half-century, yet a general mechanism that applies to most proteins remains missing^10^. The earliest classic view that proteins fold non-randomly through intermediate states on “predetermined” folding pathways, came from the famous Levinthal paradox that was proposed in 1969^11^. Later studies have well demonstrated the existence of folding intermediates, on-pathway and off-pathway. Different models such as the framework model^12^, the diffusion-collision^13^, and the jigsaw puzzle model^14^ were proposed based on experimental and theoretical studies. However, a consensus has not been reached yet; some models were even contradictory to each other. The “folding funnel energy landscape” view, proposed in the 1990’s, emphasizes the ensemble nature of protein conformations, describing macroscopic “states” as a distribution of various conformations during parallel folding events^15^. In this “funnel” theory, the so-called off-pathway states are energy traps that are direct and “on-route” for the population involved in these pathways. In more recent years, different hierarchical hypotheses have been revived, such as the “zipping and assembly”^16^ and “foldon” views^3,17–20^. The concept of “foldon”, small cooperative folding unit, emerged since the folding of many proteins was detected to be sub-globally cooperative. The “foldon” view embraces the idea of predetermined macroscopic folding pathways and suggests the sequential nature of “foldon” forming processes.

In most of the views, folding intermediates are the keys to understand the folding processes. Although they have been detected in many protein systems, obtaining their specific structural information has been challenging, as high energy intermediate states are often transiently formed and sparsely populated in the native unfolding-folding equilibrium^21^. The situation is changing now. The advancement of powerful analytical techniques in recent years such as nuclear magnetic resonance (NMR)^3,22–24^ and mass spectrometry^9^ has made it possible to characterize the intermediate states at high temporal and spatial resolution.

Shifting protein folding equilibrium by co-solvents such as heavy water (D_2_O) and urea has been well characterized in the literature. Most of the protein molecules are more stable (Table S1) and more rigid^25^ and fold more rapidly^26^ in D_2_O than in H_2_O, because of stronger D-bond than H-bond in neutral water^27^. Urea is a widely used denaturant although its mechanism of denaturing remains not clearly known. Studies suggest that urea denatures proteins by a “direct interaction” mechanism, as urea has preferential binding to all regions of proteins and can form H-bond with protein backbones^28,29^.

Recently we have found that the acyl carrier protein from *Micromonospora echinospora* (*me*ACP, PDB code: 2l9f) exists in one major native folded state (N), one minor largely unfolded state (U), and one minor intermediate state (I) under native conditions^2,30^. The three states are in relatively slow conformational exchange on the sub-second timescale, following the pathway N ↔ U ↔ I or U ↔ N ↔ I. Beyond this, the overall structural features of this state I were mostly uncertain. In this study, we deepened our study of *me*ACP folding to explore structural features of state I using chemical exchange saturation transfer (CEST) NMR. We extracted the backbone ^13^Ca chemical shifts of *me*ACP in the state I, and demonstrated that this state is a partially unfolded form. In an effort to understand the folding dynamics thoroughly, the folding equilibria were compared under varied conditions: D_2_O, H_2_O, and low concentration of urea (0.25 M and 0.5 M). The comparison provides insights into the folding of *me*ACP.

## Results and Discussions

**Figure 1:**
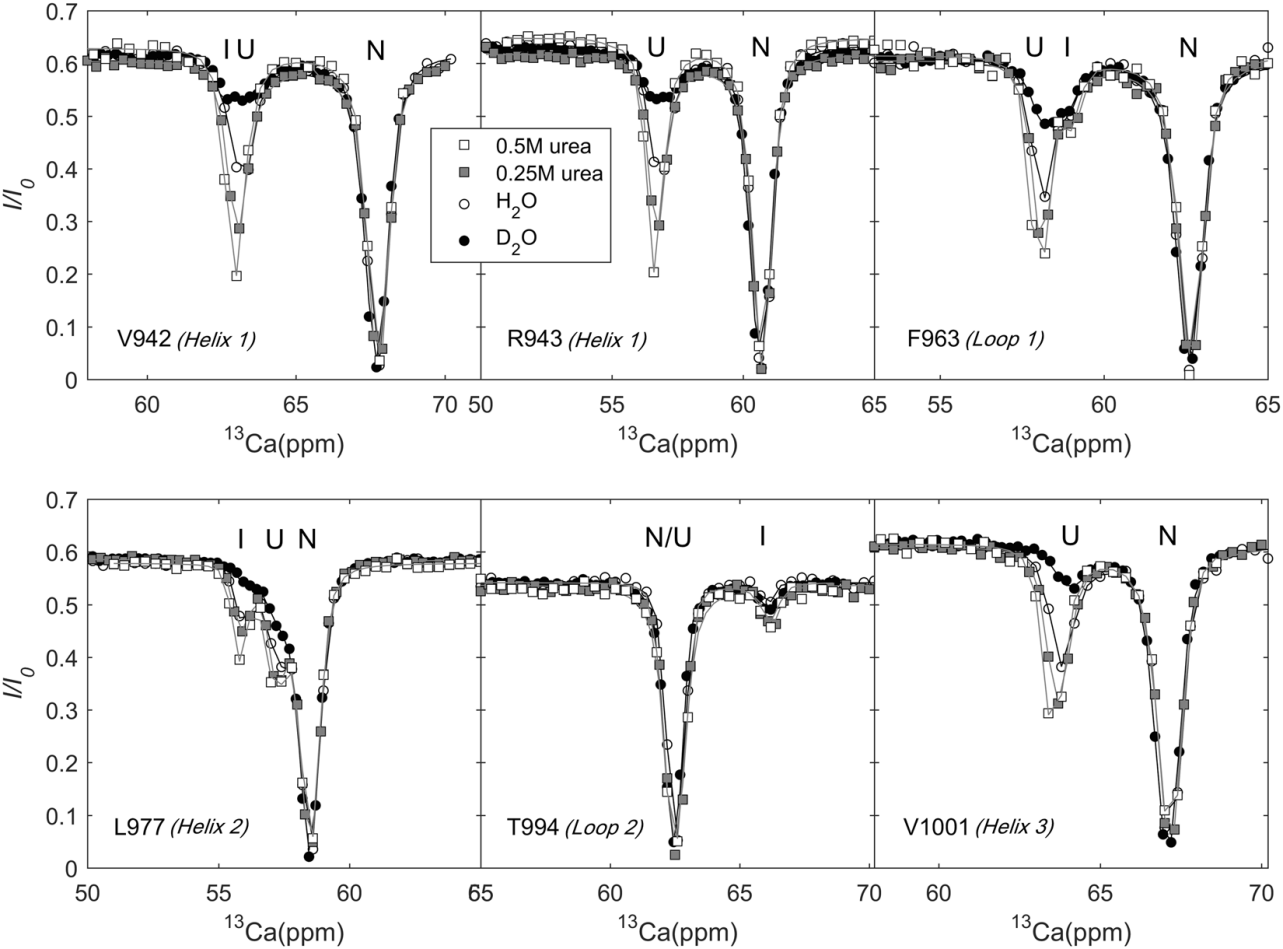
Representative ^13^Ca CEST profiles obtained with a RF weak field of 15 Hz in H_2_O, D_2_O, 0.25 M urea, and 0.5 M urea. The data obtained with a field of 30 Hz are not included for visual clarity. Solid lines are the best fits.

^13^Ca and ^15^N CEST experiments on *me*ACP in H_2_O, D_2_O, H_2_O + 0.25 M urea, and H_2_O + 0.5 M urea were performed. 43 residues displayed two dips in their ^13^Ca CEST profiles, showing those residues existed in at least two forms. As demonstrated previously on the basis of chemical shifts, the minor form was mainly unfolded (U) with α-helical propensity^2,31^. Moreover, a third state (I) was clearly seen in their CEST profiles for several residues (Figs. 1 and S1).

**Figure 2:**
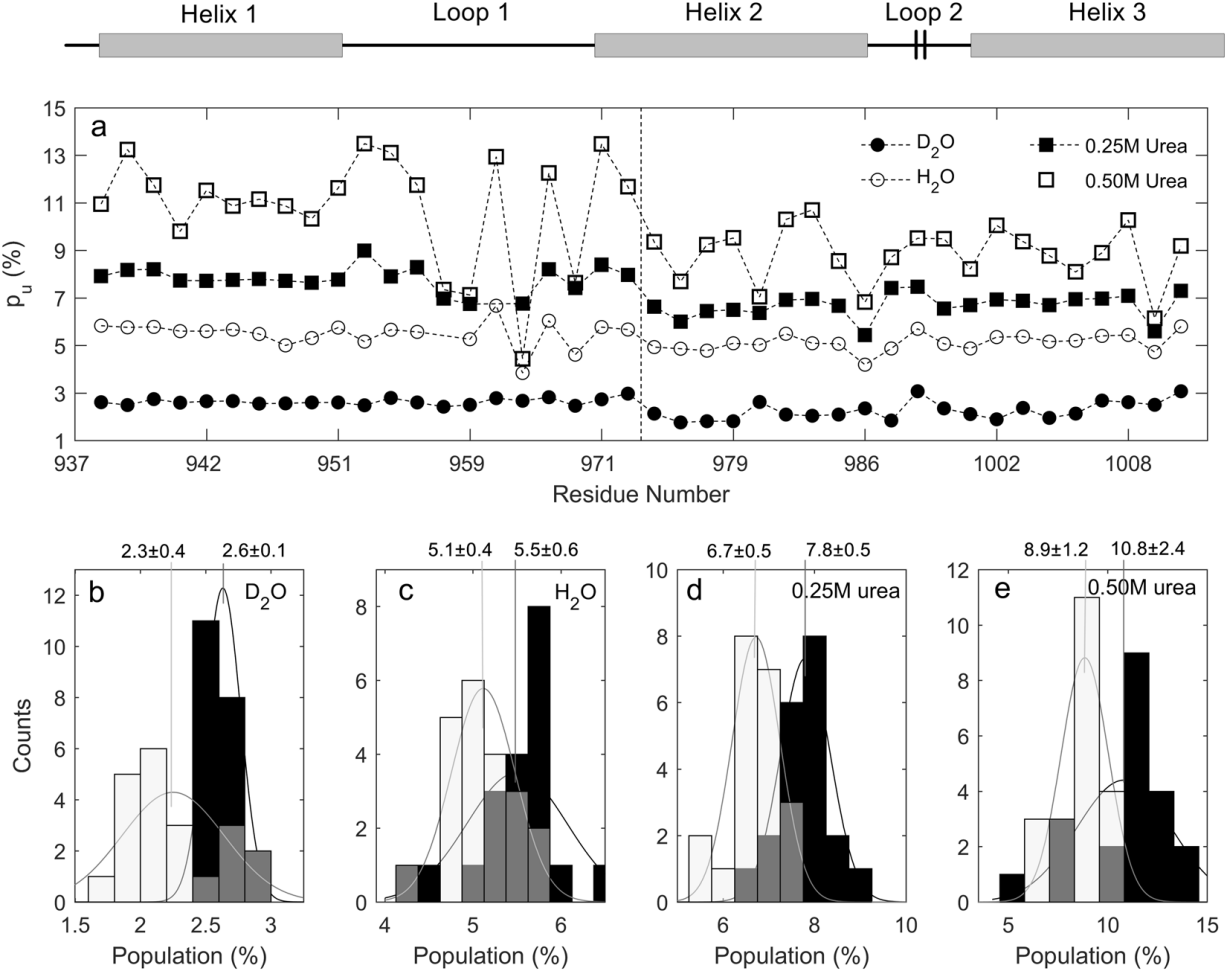
Population of state U *p*_*u*_ (**a**), *p*_*u*_ distribution of the two half regions (N-terminal: black bar; C-terminal: white bar; overlapped portion of black and white bars: grey bar) of *me*ACP in D_2_O (**b**), H_2_O (**c**), 0.25 M urea (**d**) and 0.5 M urea (**e**). The averages and standard deviations of p_u_ for the two halves in different solvents are shown on the top of each panel.

For the residues displaying two-dip CEST profiles, we first performed individual fitting using the two-state model. The population values of state U (*p*_*u*_) extracted from the ^13^Ca CEST are shown in Fig. 2. The results from the ^15^N CEST are given in Fig. S4. Interestingly, the average *p*_*u*_ values obtained by individual fitting with the two-state model for the residues in the C-terminal half region (C-terminal region of helix 2 and helix 3: from V974 to A1010) were smaller than those for the residues in the N-terminal half region (helix 1, loop 1 and N-terminal region of helix 2: from A938 to I972). The t-tests (p-value < 1.4% for all data sets) confirmed that the differences were significant. The population differences between the two regions showed that the folding of *me*ACP was not globally all or none. Is there a partially folded state where the C-terminal region remains folded while the N-terminal region is unfolded?

When the dip for state I overlaps with that for state U (i.e., ${{\rm{\Omega }}}_{U}\approx {{\rm{\Omega }}}_{I}$) or overlaps with that for state N (${{\rm{\Omega }}}_{N}\approx {{\rm{\Omega }}}_{I}$), the CEST profiles of a three-state system look like two-state profiles, but cannot be fitted well to the global two-state model. The bad global fitting of our data by the two-state model indicates the global presence of state I (Fig. S2). In addition, 18 (out of 93) residues displayed three-dip ^15^N CEST profiles with clear evidence of a third state I^2^. Moreover, the dip depth for state I in CEST profiles increased with urea concentration (Figs. 1 and S1), and the difference of *p*_*u*_ values between the N- and C-terminal regions increased as well (Fig. 2). Taken together, we suggest that the difference of *p*_*u*_ between the two regions shown in Fig. 2 comes from the global presence of state I.

**Figure 3:**
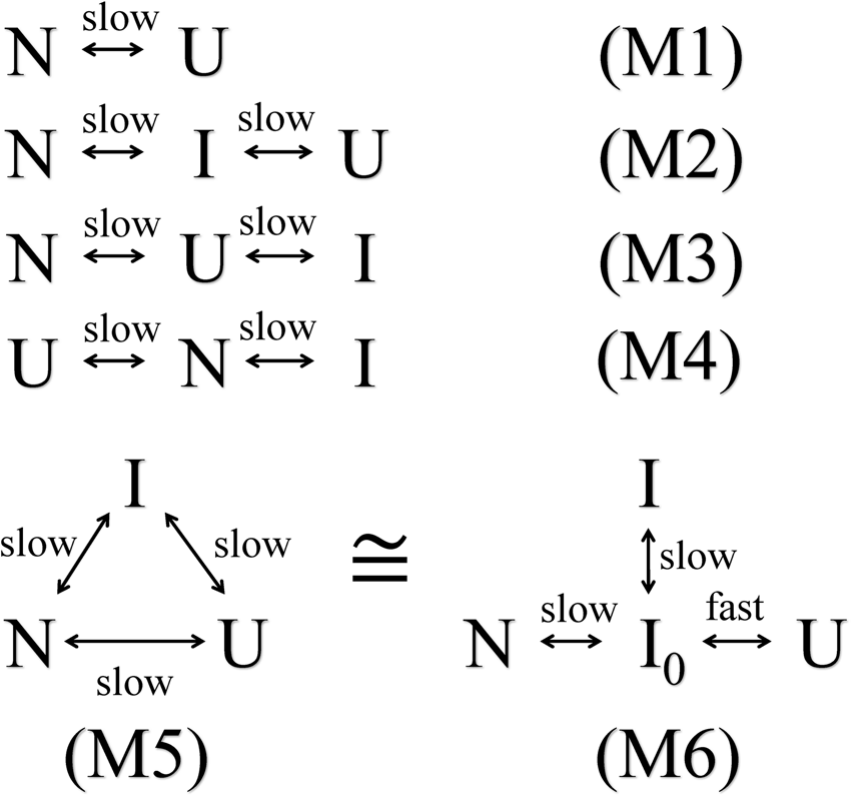
Possible exchange models examined for *me*ACP.

For a system with three conformational states, there are four possible three-state exchange models (M2~M5 in Fig. 3). As discussed in our previous paper^2^, the exchange rates between N and U and between N and I were around 250 s^−1^ and 100 s^−1^, respectively, and model M2 was excluded based on the ^15^N data. In the on-pathway model (M2 in Fig. 3), the exchange between N and I must be faster than the exchange between N and U, which is contradictory to the result derived from the ^15^N data. It is noteworthy that models M3 and M4 are two special cases of M5, where the exchange rate between I and N or the rate between I and U is zero.

For each data set, global fittings based on the two-state model and three-state models were conducted to compare the fitting goodness, and to extract the global exchange rates and populations of different states (shared by all residues) as well as the per-residue chemical shifts of minor states (Ω_*U*_ only in two-state model; Ω_*U*_ and Ω_*I*_ in three-state models), transverse relaxation rates (R_2*N*_) and longitudinal relaxation rates (R_1*N*_) of the major state. The reduced *x* ^2^ values for global fittings by three possible three-state models M3~M5 were significantly smaller than those for the two-state model M1 and the on-pathway three-state model M2 (see Table S2). The F-tests showed that the global three-state models M3~M5 described the CEST data significantly better than models M1 and M2 (p-value < 10^−4^ for all data sets). Indeed, the two-state model failed obviously for a few residues that displayed three dips. Considering the possibility that global minima might not be reached in the global fittings by the three-state models (due to large search space) and the fact that the fitting residuals by M3~M5 were similar, we think that the models M3~M5 matched equally well with the data (Table S2). So we could not determine which model is true for *me*ACP. Fortunately, the ^13^Ca chemical shifts of state I (Ω_*I*_) obtained with these three three-state models were almost identical.

As the Ω_*I*_ values were extracted by minimizing χ^2^, it is likely that Ω_*I*_ could be positioned incorrectly at Ω_*U*_ in a few cases when Ω_*I*_ and Ω_*U*_ were very similar and χ^2^ values were not sensitive to Ω_*I*_ positions. Nevertheless, Ω_*I*_ could be determined at high confidence level from the χ^2^ distribution for most residues.

### Structural features of the two minor states

**Figure 4:**
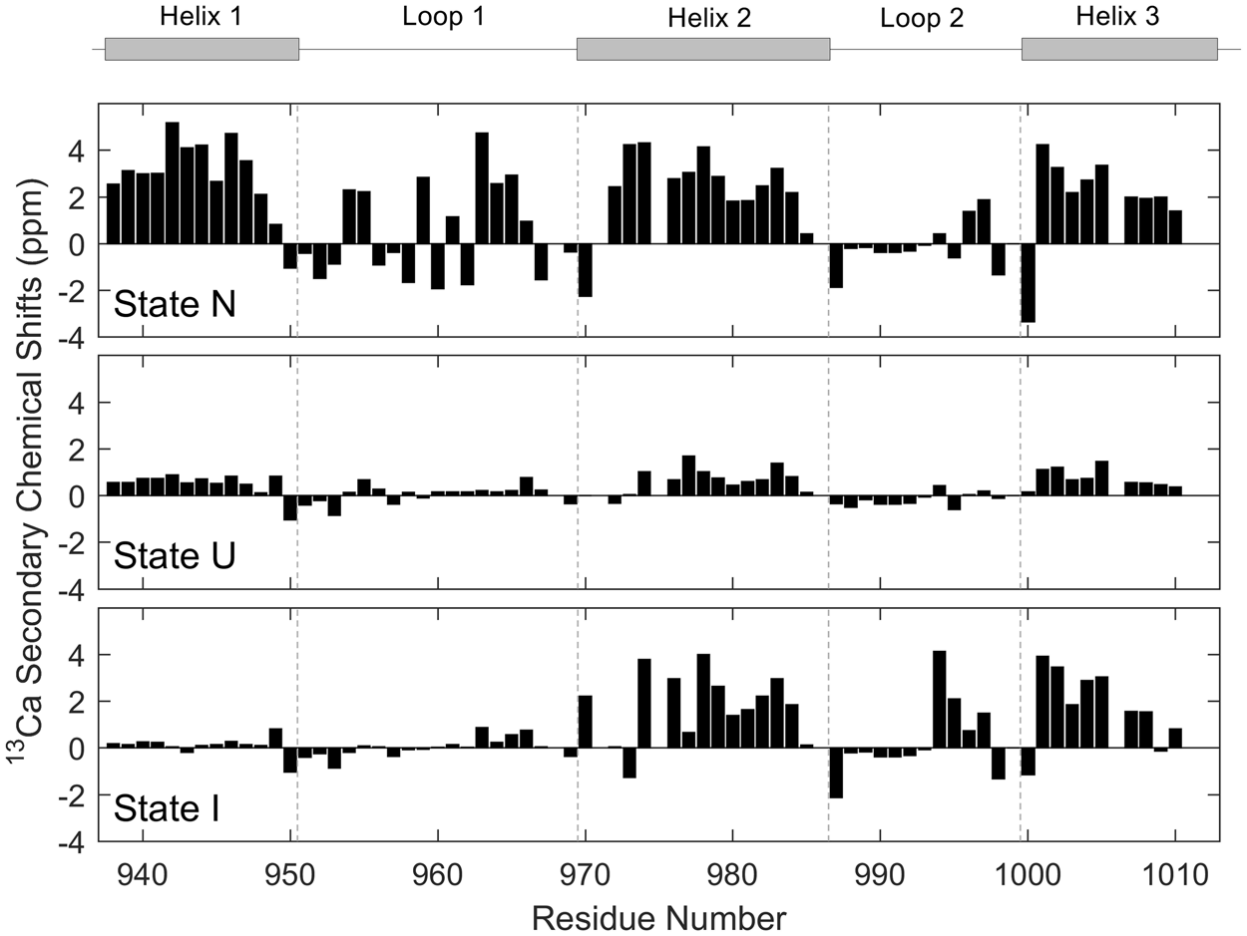
^13^Ca secondary chemical shifts of state N, U and I in D_2_O. ^13^Ca chemical shifts experimentally measured in 4 M urea (fully denatured) were used as the reference.

The secondary chemical shifts (CSs) of each residue in three states were calculated by subtracting the random coil chemical shift (CS) values, which were experimentally measured in the fully denatured sample (*me*ACP in 4 M urea), from the CS values of the corresponding residues which were obtained in the CEST data. The analysis of ^13^Ca secondary CSs is a simple method to identify the secondary structures of a protein in different states^32^ (Fig. 4).

For state U, the ^13^Ca secondary CSs in three helical regions had an average value of around +1 ppm, as compared with state N whose ^13^Ca secondary CSs in the helical region were in the range of +2~+3 ppm (Fig. 4), indicating the presence of residual helical structures. The residual helical pattern in state U was the same as that in the native state, implying that the three native helices were not fully unfolded in state U. The ^13^Ca secondary CS amplitudes for the helical regions in state U were about one-third of those in state N, suggesting that the three helices in state U are populated for about 30% of the time.

For state I, the ^13^Ca secondary CSs of its N-terminal region (helix 1, loop 1, and N-terminal region of helix 2) were close to zero, indicating this region exists in a nearly fully unfolded form. On the other hand, the ^13^Ca secondary CSs of the C-terminal region (helix 3 and C-terminal region of helix 2) in state I were mostly similar to those in state N, showing that the C-terminal region of state I is native-like (Fig. 4) (or the helices 2 and 3 remain mainly folded in state I).

Obviously, the state I is overall a partially unfolded form (PUF). This is consistent with the results shown in Fig. 2. Based on the two-state model, a residue can stay as either a folded or an unfolded form. As the N-terminal region has a higher unfolded population than the C-terminal region (Fig. 2), some protein molecules must exist as a PUF. Based on ^15^N CEST data alone, we previously suggested that state I adopted a locally altered conformation in which the N-terminal region of helix 2 differs from both helical and random coil structures while other helices are native-like^2^. Based on ^13^Ca and ^15^N data, it is now clear that the state I resembles state N in the C-terminal half, while it resembles state U in the N-terminal half (Fig. 4). It is noteworthy that state I still differs significantly from state N in the N-terminal region of helix 2.

### The partially unfolded form (PUF or state I)

In one scenario, the folding follows the triangle model (M5 in Fig. 3). It might suggest parallel folding pathways. The terms *p*_*I*_ * *k*_*IN*_ and *p*_*u*_ * *k*_*UN*_ can represent the contributions to folding from apparent folding pathways U-I-N and U-N, respectively. $\frac{{p}_{I}\ast {k}_{IN}}{{p}_{u}\ast {k}_{UN}}$ was estimated to be smaller than ~0.06 for all data sets (Table S3), showing that the pathway U-I-N contributes significantly less than the U-N pathway in the protein folding. Although the triangle model fits the experimental data, it is not necessary for the folding to must be parallel, because a four-state model with state I as an off-pathway product (M6 in Fig. 3) still fits the data. Therefore, the state I might be an “on-pathway” folding intermediate state and it is also possible to be an “off-pathway” intermediate state.

The triangle model can be approximated as the single-pathway model N-U-I (M3) when ***k***_*IN*_ + ***k***_*NI*_ is much smaller than ***k***_***IU***_ + ***k***_***UI***_. It can also be approximated as the model U-N-I (M4) when ***k***_*IN*_ + ***k***_*NI*_ is much larger than ***k***_***IU***_ + ***k***_***UI***_. For some of our data sets ***k***_*IN*_ + ***k***_*NI*_ ≫ ***k***_***IU***_ + ***k***_***UI***_, and for other data sets ***k***_***IN***_ + ***k***_***NI***_ ≪ ***k***_***IU***_ + ***k***_***UI***_ (Table S3). This is caused by the fact that each set of data could be fitted equally well by M3 and M4 (Table S2). Because the folding can follow the two possible single-pathway models, state I might be an “off-pathway” state of the folding from state U to N. We previously suggested that there might be a dynamic equilibrium between monomeric and oligomeric forms, and the oligomers might result from this state I^2^. The N-terminal region of helix 2 (around S970-L977) in state I is very non-native (Fig. 4), which might be prone to aggregate. The misfolding of this region might cause the failure of the further folding of state I.

If state I_0_ is on the folding path from U to I_0_ to N, but local misfolding happens for a fraction of this state and blocks its folding to state N due to “the occurrence of an on-pathway optional error”^33^, the misfolded fraction (state I) appears as an off-pathway product (“dead-end”) (M6 in Fig. 3). Therefore, the “off-pathway” state I may represent the misfolded population, which is only a fraction of the constructive state I_0_ with optional local misfolding errors. This putative “on-pathway” intermediate state I_0_ would be similar to the observed state I in overall structure, but it would interconvert with state U so fast that it could not be detected by CEST and relaxation dispersion.

### Comparison of folding dynamics in D2O, H2O, and urea

The effects of co-solvent D_2_O and urea on shifting the folding equilibria were examined. The ^13^Ca CEST profiles recorded in H_2_O, D_2_O and urea varied mainly in the depths of the minor dips (Fig. 1). The dips for both states U and I were larger in urea and smaller in D_2_O, compared with those in H_2_O (see Fig. 1 and Fig. S1).

Table 1 shows the extracted folding parameters in D_2_O, H_2_O, 0.25 M urea and 0.50 M urea by following M3 (N-U-I). Models M4 and M5 gave similar *p*_*u*_, *k*_*UN*_, *k*_*NU*_, Ω_*U*_, and Ω_*I*_ values as M3 did (see Tables S3 and S4). As buffer condition became more denaturing (from D_2_O to H_2_O, to higher concentration of urea), the population of unfolded state (*p*_*u*_ **)** increased, reflecting the decrease of Gibbs energy of unfolding (ΔG_*N*–*U*_). We do expect unfolded states (U) to be more populated in urea, less in D_2_O, as D_2_O and urea have been widely known for their stabilizing and destabilizing effects on proteins, respectively. *me*ACP was ~0.5 kcal/mol more stable in D_2_O (Table 1). This is consistent with literature studies (summarized in Table S1). Previous data suggest that the larger the proteins are, the more prominent the stabilizing effect is (Fig. S3). The stability enhanced by D_2_O agrees well with the size of *me*ACP.

**Table 1:** Folding and unfolding based on three-state model N-U-I.

Solvents	Expt.	p_u_ (%)	p_I_ (%)	ΔG_NU_ (kcal/mol)	ΔG_NI_ (kcal/mol)	k_NU_ (s^−1^)	k_UN_ (s^−1^)	k_UI_ + k_IU_ (s^−1^)
D_2_O	^13^Ca	2.0 ± 0.01	~2.0*	2.3	~2.3*	10.4 ± 2.3	495.2 ± 7.9	~73*
H_2_O	^13^Ca	4.6 ± 0.02	~1.9	1.8	~2.3	16.9 ± 2.6	356.6 ± 10.4	~141
H_2_O	^15^N	4.8 ± 0.01	~0.8	1.8	~2.8	15.0 ± 0.3	297.6 ± 4.9	~85
0.25 M Urea + H_2_O	^13^Ca	6.5 ± 0.04	~2.1	1.6	~2.2	14.9 ± 0.8	220.0 ± 5.5	~93
0.25 M Urea + H_2_O	^15^N	6.9 ± 0.05	~6.9	1.5	~1.5	15.7 ± 0.3	199.6 ± 2.7	~34
0.50 M Urea + H_2_O	^13^Ca	7.9 ± 0.04	~8.5	1.4	~1.4	15.6 ± 0.6	176.5 ± 4.1	~31

^*^, “~” is used to indicate that the results were estimated as *p*_*I*_ and (***k***_***UI***_ + ***k***_***IU***_) are correlated in the fitting.

The exchange between states N and U can be viewed as the major folding/unfolding process, therefore the conversion rates from U to N (*k*_*UN*_) and from N to U (*k*_*NU*_) were denoted as the folding (*k*_*f*_) and unfolding (*k*_*u*_) rates, respectively. According to the results in Table 1, Tables S3 and S4, *k*_*f*_ (*k*_*UN*_) decreased as solvent became more denaturing; *k*_*u*_ (*k*_*NU*_) increased when solvent was changed from D_2_O to H_2_O, and there were no significant effects on *k*_*u*_ by a low concentration of urea.

There were large uncertainties in the population (*p*_*I*_) and exchange rates of state I (*k*_*X*–*I*_ and *k*_*I*–*X*_, which are the exchange rates between I and N and between I and U) because the fitting *x* ^2^ values changed little when *p*_*I*_, *k*_*X*–*I*_, and *k*_*I*–*X*_ were within certain ranges. Therefore, we only estimated *p*_*I*_ and the sum of *k*_*X*–*I*_ and *k*_*I*–*X*_. Despite the large uncertainty in the estimated *p*_*I*_, we observed in the raw profiles that the dips for state I were larger in urea, and smaller in D_2_O than in H_2_O (Figs. 1 and S1). The variations of state I dips in the raw profiles indicate that the population of state I increases with the destabilization strength of the solvent (in current range of low concentrations of urea).

### Effects of urea on *me*ACP folding and unfolding

High concentration of urea is denaturing, while the effects of low concentration of urea on protein stability seem to vary for different proteins and solvent environments. It was shown that low concentration urea stabilizes protein ferrocytochrome c^34^. Here we found that *me*ACP was destabilized, even at the concentration of urea as low as 0.25 M. The populations of both state U and state I increased with urea concentration.

According to Eq. 4 (see Materials and Methods section), the effect of urea on *k*_*f*_ mainly originates from the change of energy barrier (activation energy ${G}^{\ddagger }$), because the changes of viscosity and internal friction at low concentration of urea buffer (0.25 M) are negligible ($\approx 1 \%$)^35,36^. Therefore, the change of activation energy is given by,

1 $${\rm{\Delta }}{\rm{\Delta }}{G}_{urea-H2O}^{\ddagger ,f}={\rm{\Delta }}{G}_{urea}^{\ddagger ,f}-{\rm{\Delta }}{G}_{H2O}^{\ddagger ,f}\approx -RTln(\frac{{k}_{f}^{urea}}{{k}_{f}^{H2O}})$$

**Figure 5:**
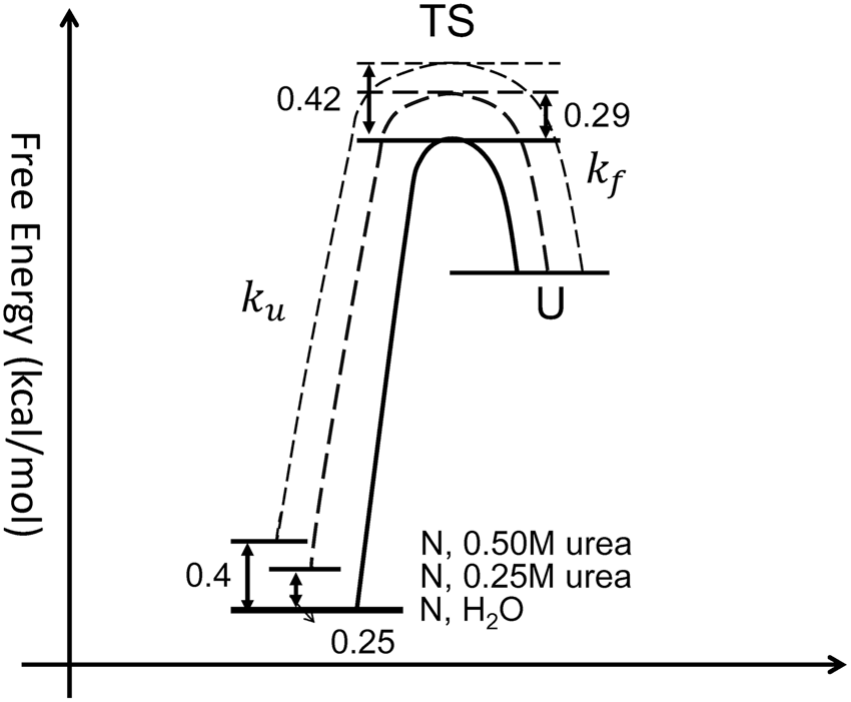
Schematic plot of relative free energy profiles of meACP folding in varied solvents. The free energies in states TS and N are relative to that in state U. (Energy values were calculated based on Table 1).

Using the results in Table 1, ${\rm{\Delta }}{\rm{\Delta }}{G}_{urea-H2O}^{\ddagger ,f}$ was estimated as ~0.29 kcal/mol and ~0.42 kcal/mol for *me*ACP at 0.25 M and 0.5 M urea, respectively (Fig. 5). Similarly, ${\rm{\Delta }}{\rm{\Delta }}{G}_{urea-H2O}^{UN}$ (the change of the Gibbs energy of folding, ${\rm{\Delta }}{G}_{urea}^{UN}-{\rm{\Delta }}{G}_{H2O}^{UN}$) was calculated from extracted *p*_*u*_ values with Eq. 3. ${\rm{\Delta }}{\rm{\Delta }}{G}_{urea-H2O}^{UN}$ was ~0.25 kcal/mol and ~0.40 kcal/mol for *me*ACP in 0.25 M and 0.5 M urea (relative to H_2_O), respectively (Fig. 5). Accordingly,

2 $${\varphi }_{f}=\frac{{\rm{\Delta }}{\rm{\Delta }}{G}_{urea-H2O}^{\ddagger ,f}}{{\rm{\Delta }}{\rm{\Delta }}{G}_{urea-H2O}^{UN}}\approx 1$$

The *ϕ*_*f*_ factor, or so-called Tanford’s *β* value, is viewed as an indicator of the average degree of changes in accessible surface areas (Δ*ASA*) of the transition state (TS) relative to that of the unfolded state during the protein folding^37^. *ϕ*_*f*_, generally ranging from 0 to 1, represents the ratio of TS stability change to native state (N) stability change. A *ϕ*_*f*_ value of 1, meaning that the relative stability change of TS equals to that of N, suggests that the overall Δ*ASA* of TS and that of N are similar during unfolding. Thus, ${\varphi }_{f}\,\approx 1$ indicates that the TS may have a native-like structure. According to the results obtained in D_2_O, we found that *ϕ*_*f*_ > 1/2, further supporting that the TS is native-like (see discussion in the supplementary information).

Overall, the results here suggest that the TS could be placed close to state N on the path N ↔ U in the possible models (M3~M6) shown in Fig. 3. In M3, the pathway would be N ↔ TS ↔ U ↔ I when considering the TS; in M4 it would be U ↔ TS ↔ N ↔ I; in M5 it would be N ( ↔ I) ↔ TS ↔ U ( ↔ I); in M6 it would be U ↔ I_0_ ( ↔ I) ↔ TS ↔ N, according to their structural features (or overall folded percentages). The TS is suggested to be a folded transition state that is close to the native state, sharing a similar structure with the native state.

## Conclusion

We found that there exists a partially unfolded form of *me*ACP, where the N-terminal half is unfolded while the C-end half remains mainly folded. The existence of PUF was confirmed by ^15^N and ^13^Ca CEST results in different solvents. The detected PUF could be either an “on-pathway” intermediate state in the triangle model or an “off-pathway” state in the other three-state models and four-state model. This PUF might represent a fraction of the constructive folding intermediate, and occurred to be misfolded. The structure features of this PUF might suggest the sequential folding order in which the C-terminal region folds before N-terminal region does. Furthermore, the H/D isotope effect and urea effect on *me*ACP folding dynamics were investigated by comparing CEST results in different solvent conditions. Consistent with literature studies on H/D isotope effects, the native state of *me*ACP was more stable in D_2_O, folding was accelerated, and unfolding was decelerated compared to that in H_2_O. Low concentration of urea destabilized the native state of the protein and seemed to stabilize state PUF. It also slowed down the folding rates while had no effects on the unfolding rates, suggesting the structural similarity between the transition state and native state of *me*ACP.

## Materials and Methods

### Sample preparation and NMR spectroscopy

Different acyl carrier protein samples (Table 2) were prepared by following previous protocols^38^. All NMR samples contained 0.6~1 mM protein, 50 mM NaCl, 5 mM EDTA, and 50 mM phosphate at pH 6.9. ^13^Ca CEST and ^15^N CEST experiments were performed at 25 °C on a Bruker 800 MHz NMR spectrometer equipped with a cryoprobe. The experimental parameters used were the same as those described previously^2,31^.

**Table 2:** CEST experiments.

No.	Sample labeling	Solvent Condition	Experiment
1	^13^C	95% H_2_O, 5% D_2_O	^13^Ca CEST
2	^13^C	100% D_2_O	^13^Ca CEST
3	^13^C	95% H_2_O, 5% D_2_O + 0.25 M urea	^13^Ca CEST
4	^13^C	95% H_2_O, 5% D_2_O + 0.50 M urea	^13^Ca CEST
5	^15^N	95% H_2_O, 5% D_2_O	^15^N CEST
6	^15^N	95% H_2_O, 5% D_2_O + 0.25 M urea	^15^N CEST

### CEST data analysis

#### Individual and global fitting using the two-state model

The residues displaying two dips separated by more than 1 ppm were chosen to extract kinetics parameters. Their CEST profiles were first fitted individually using the two-state model (M1 in Fig. 3) to obtain parameters for each residue, i.e., folding and unfolding rates (*k*_*f*_, *k*_*u*_), population of the minor state (*p*_*u*_), chemical shift in the minor state (Ω_*U*_), longitudinal relaxation rates of major (R_1*N*_) and minor states (R_1*U*_), transverse relaxation rates of the major (R_2*N*_) and minor states (R_2*U*_). In the fitting, the J coupling effect was taken into account as described previously^39^. We assumed that ^1^J_COCα_ = 55 Hz and ^1^J_CαCβ_ = 35 Hz for all residues in ^13^C labelled protein samples; and set R_1*U*_  = R_1*N*_ for each residue. When R_2U_ was used as an independent fitting parameter, the extracted R_2U_ for most residues deviated significantly from the expected values for our system due to the presence of a third state that overlaps with state N or U as well as deviations of ^1^J_COCα_ and ^1^J_CαCβ_ from the assumed values. To simplify the fitting, we assumed all the residues have the same R_2U_ values in the unfolded state and set R_2*U*_ = 6.5 and 8.1 s^−1^ for ^13^Ca in H_2_O and D_2_O respectively and 3.0 s^−1^ for ^15^N in H_2_O at 25 °C. These values were estimated based on the ^15^N R_2_ values of intrinsically disordered protein α-synuclein (~3.5 s^−1^), which were measured at 15 °C on an 800 MHz spectrometer^40^. The same assumption was made for three-state fittings. In global fitting, all residues shared a common exchange rate (*k*_*ex*_) and a common population of the minor state (*p*_*u*_), but they each had unique *R*_1*N*_, *R*_2*N*_, and Ω_*U*_ values.

#### Individual fitting and global fitting using three-state models

The procedures for fitting the CEST data of individual residues to three-state models (M2 ~ M5) were the same as that described above. The residues displaying two and three dips were also fitted globally to the three-state models. In the fitting, we set *R*_2*I*_ = *R*_2*N*_ and *R*_1*I*_ = *R*_1*U*_ = *R*_1*N*_ for each residue, and assumed ^1^J_COCα_ = 55 Hz and ^1^J_CαCβ_ = 35 Hz for all residues. For residues displaying three well separated dips, Ω_*U*_ and Ω_*I*_ values were already certainly known. For residues displaying two-dip profiles, Ω_*U*_ values were quite certain and Ω_*I*_ should be close to either Ω_*N*_ or Ω_*U*_. Optimization was done by extensive grid-search of Ω_*I*_. To extract the Ω_*I*_ value as accurate as possible, we used the following procedure:

- The Ω_*I*_ of each residue was first estimated by individual fitting via grid search of Ω_*I*_;
- Ω_*I*_ values obtained in step *a* were used as the input values in the global fitting to obtain global exchange rates between states N and U (k_1_) and between I and U or I and N (k_2_), populations of states N (*p*_*N*_), and U (*p*_*u*_);
- Ω_*I*_ values were re-calculated by individual fitting with fixed global k_1_, k_2_, *p*_*N*_, and *p*_*u*_ values (obtained in step *b*);
- Repeat steps *b* and *c*, until χ^2^ of the global fitting decreased to a stable value.

#### Error estimation

For each CEST profile, the uncertainty (δ) in intensity ratio (***I/*** *I*_0_) was estimated by calculating the standard deviation of data points in the ‘non-saturation area’. The following Monte Carlo simulations were used to estimate fitting errors of extracted CEST parameters for each residue:

- Generate 120 sets of profiles using extracted parameters, add random noise with a standard deviation of δ and mean of 0;
- The 120 set of profiles were fitted to extract 120 sets of fitting parameters. The standard deviation of each parameter was considered as the fitting error.

To determine global fitting errors, 80% of the residues used in the global fitting were randomly taken to extract global parameters and repeat 120 times to obtain standard deviations.

#### Energy calculations

According to the population of each state, the Gibbs energy change (ΔG) of process A to B was calculated by

3 $${\rm{\Delta }}{G}_{AB}=-RT\,\mathrm{ln}({p}_{B}/{p}_{A})$$

where *p*_*A*_ and *p*_*B*_ are the populations of states A and B, respectively. The subscript AB is short for A → B.

The folding rate (*k*_*f*_) is related to the activation energy (${G}^{\ddagger }$) by^41,42^

4 $${k}_{f}=C{(\sigma +\eta )}^{-1}{e}^{-{\rm{\Delta }}{G}^{\ddagger }/RT}$$

where C is the frequency factor for the folding process, *σ* refers to the “internal friction”, and *η* is the viscosity of solvent. *σ* reflects the contribution of the energy landscape ruggedness to the reaction rate and is dominated by the structure of a protein when denaturant concentrations are low^36^.

### Data Availability

The datasets used in the current study are available in the supplementary information file, and the Matlab scripts for data fitting are available from the corresponding author upon request.

## Electronic supplementary material

Supplementary Information

Supplementary data
