# Supplementary material for: Equilibrium folding dynamics of meACP in water, heavy water, and low concentration of urea
Source: Sci Rep. 2017 Nov 23;7:16156. doi: 10.1038/s41598-017-16449-4 (PMC5700953; doi:10.1038/s41598-017-16449-4)
Supplement: Supplementary file 1 — Supplementary Information [file 41598_2017_16449_MOESM1_ESM.pdf]

Supplementary Information

Equilibrium folding dynamics of *me*ACP in water, heavy water, and low concentration of urea

Yang Zhou and Daiwen. Yang*

Department of Biological Sciences, National University of Singapore, 14 Science Drive 4, 117543, Singapore

Discussion on H/D isotope effects

The H/D isotope effects measured here on protein stability and folding rates have two origins: the replacement of amide H by D (amide isotope effect) and replacement of solvent (solvent isotope effect). The amide isotope effect is caused by the difference of H-bonding and D-bonding associated with backbone NH and CO moieties. This effect destabilizes α-helical proteins but is negligible to β-sheet proteins[1]. The extent of destabilization to a protein is correlated with the number of helical hydrogen bonds in the protein. On the other hand, the solvent isotope effect has been shown to stabilize a protein[2,3], which is independent of helical hydrogen bonding. As the D-bond of water is 0.1-0.2 kcal/mol more stable than the H-bond[4], the deuterium water provides a more rigid and compacting environment for protein, enhancing the hydrophobic interactions noticeably through the solvent isotope effect. If the amide isotope effect contributes more significantly to the stability, we expect protein is less stable in D_2O since *me*ACP is a helical protein[5]. *me*ACP was ~0.5 kcal/mol more stable in D_2O (Tab. 1), showing the solvent isotope effect was more prominent.

According to Eq. 4 in the main text (see Materials and Methods section), the H/D solvent effects on k_f (k_{UN}) and k_u (k_{NU}) have two origins: energy barrier changes induced by co-solvents (changes of $\Delta G^{\ddagger}$) and "viscosity-specific" effects (η). As changing buffer condition from H_2O to D_2O, η increases from ~0.89 mPa·s to ~1.10 mPa·s at 25 ºC and $\Delta G_f^{\ddagger}$ is expected to be changed. The decrease (or increase) of $\Delta G_f^{\ddagger}$ is expected to accelerate (or decelerate) k_f, and the increase of η is expected to slow down k_f. Obviously the decrease of $\Delta G_f^{\ddagger}$ plays a dominating role as our results show a significantly accelerated folding (Tab.

1, Tabs S3 and S4). From the change of k_f, we can infer the change of $\Delta G_f^{\ddagger}$ ($\Delta\Delta G_{D20-H20}^{\ddagger,f} = \Delta G_{D2O}^{\ddagger,f} - \Delta G_{H2O}^{\ddagger,f}$):

$$\frac{k_f^{D2O}}{k_f^{H2O}} = \frac{C(\sigma + \eta_{D2O})^{-1} e^{-\Delta G_{D2O}^{\ddagger,f}/RT}}{C(\sigma + \eta_{H2O})^{-1} e^{-\Delta G_{H2O}^{\ddagger,f}/RT}} = \frac{(\sigma + \eta_{H2O})}{(\sigma + \eta_{D2O})} e^{-(\Delta G_{D2O}^{\ddagger,f} - \Delta G_{H2O}^{\ddagger,f})/RT}$$

$$< e^{-(\Delta G_{D2O}^{\ddagger,f} - \Delta G_{H2O}^{\ddagger,f})/RT} \quad [S1]$$

From Eq. S1, we have

$$\Delta G_{D2O}^{\ddagger,f} - \Delta G_{H2O}^{\ddagger,f} < -\mathrm{RT}\ln\left(\frac{k_f^{D2O}}{k_f^{H2O}}\right) \approx -0.25\ kcal/mol \quad [S2]$$

According to Tab.1, $\Delta G_f^{D2O} - \Delta G_f^{H2O} = -(\Delta G_{NU}^{D2O} - \Delta G_{NU}^{H2O}) = -0.5\ kcal/mol$, and then

$$\frac{\Delta G_{D2O}^{\ddagger,f} - \Delta G_{H2O}^{\ddagger,f}}{\Delta G_f^{D2O} - \Delta G_f^{H2O}} > \frac{1}{2} \quad [S3]$$

The left part of Eq. S3 is defined as ϕ_f, following the principle of the phi-value analysis, for substitution of solvent instead of amino acid[6]. Here $\phi_f > \frac{1}{2}$, was consistent with the results from urea effects ($\phi_f \approx 1$), suggesting that the transition state ensemble is closer (more similar) to state N than to state U.

According to $\phi_f \approx 1$, which was derived from the urea effect, we assume $\phi_f \approx 1$ (or $\phi_u \approx 0$) for unfolding in D_2O, i.e., ϕ_f is a solvent independent parameter. So, we

have $\Delta G^{\ddagger,u}_{D2O} \approx \Delta G^{\ddagger,u}_{H2O}$.

$$\frac{k_u^{D2O}}{k_u^{H2O}} = \frac{A(\sigma + \eta_{D2O})^{-1} e^{-\Delta G^{\ddagger,u}_{D2O}/RT}}{A(\sigma + \eta_{H2O})^{-1} e^{-\Delta G^{\ddagger,u}_{H2O}/RT}} \approx \frac{(\sigma + \eta_{H2O})}{(\sigma + \eta_{D2O})}\, e^{0/RT} = \frac{(\sigma + \eta_{H2O})}{(\sigma + \eta_{D2O})} \text{ . [S4]}$$

Eq. S4 demonstrates that the reduction of unfolding rates in D_2O (k_u^{D2O}) might mainly come from the viscosity effect.

Table S1. Isotope effects on different proteins.

Protein	Amide isotope effect ($\Delta G^{ND}_{D_2O} - \Delta G^{NH}_{D_2O}$) /kcal/mol	Solvent isotope effect ($\Delta G^{NH}_{D_2O} - \Delta G^{NH}_{H_2O}$) /kcal/mol	Total ($\Delta G^{ND}_{D_2O} - \Delta G^{NH}_{H_2O}$) /kcal/mol	References	PDB code	length	Volume
rat CD2	~0	-1.26	-1.26	[2]	1CDB	105	38598
RNase A	---	---	-0.7	[7]	2AAS	124	28064
RNase T1	---	---	-1.4	[7]	1BTA	89	14849
NTL9	0.21(in H_2O) 0.27(in D_2O)	-0.67(NH) -0.61(ND)	-0.40 or -1.06 (two methods)	[3,8]	2HVF	52	8749
GCN4 coil	~0.4	~ -0.5	-0.1	[6]	1ZIK	66	11007
LSZ	---	---	-1.9	[9]	1E8L	129	32821
BSA	---	---	-3.5	[9]	4F5S	600	51372

Amide isotope effect refers to the replacement of amide hydrogen NH with ND. Positive values of ($\Delta G^{ND}_{D_2O} - \Delta G^{NH}_{D_2O}$) indicate NH is more stable than ND. Solvent isotope effect ($\Delta G^{NH}_{D_2O} - \Delta G^{NH}_{H_2O}$) refers to the change of solvent from H_2O to D_2O without changing the amide hydrogen, the negative values of ($\Delta G^{NH}_{D_2O} - \Delta G^{NH}_{H_2O}$) shows proteins are more stable in D_2O.

Table S2. Reduced χ^2 using different models.

Model \ Data set	^{15}N (single labeled, in H_2O)	^{15}N (in 0.25 M Urea, H_2O)	^{13}Ca (single labeled, in D_2O)	^{13}Ca (single labeled, in H_2O)	^{13}Ca (single labeled, in 0.25 M urea)	^{13}Ca (single labeled, in 0.50 M urea)
$N \overset{slow}{\Longleftrightarrow} U$	2.311	5.791	2.153	1.942	1.642	2.709
$N \overset{slow}{\Longleftrightarrow} U \overset{slow}{\Longleftrightarrow} I$	1.074	2.083	1.287	1.821	0.904	1.768
$U \overset{slow}{\Longleftrightarrow} N \overset{slow}{\Longleftrightarrow} I$	1.127	2.145	1.244	1.902	0.969	1.757
The triangle model	0.991	2.254	1.295	1.777	0.875	2.171
$N \overset{slow}{\Longleftrightarrow} I \overset{slow}{\Longleftrightarrow} U$	5.315	8.531	2.122	3.837	1.426	2.261

Table S3. Folding and unfolding parameters extracted with the triangle model.

Solvents	Expt.	p_u (%)	p_I (%)	ΔG_{NU} (kcal/mol)	ΔG_{NI} (kcal/mol)	k_{NU} (s^{-1})	k_{UN} (s^{-1})	k_{UI}, k_{IU} (s^{-1})	k_{NI}, k_{IN} (s^{-1})
**D_2O**	^{13}Ca	1.9±0.02	~2.5*	2.3	~2.2*	10.2 ±0.1	508±9	~3, ~0.1*	~0.5, ~21*
**H_2O**	^{13}Ca	4.6±0.04	~1.9	1.8	~2.3	17.3 ±0.1	362±6	~42, ~104	~0.1, ~0.1
**H_2O**	^{15}N	4.7±0.03	~1.9	1.8	~2.3	15.3 ±0.1	308±5	~1.5, ~0.1	~0.4, ~23
**0.25 M Urea+ H_2O**	^{13}Ca	6.2±0.05	~1.6	1.6	~2.4	15.3 ±0.1	234±6	~37, ~135	~0.1, ~14
**0.25 M Urea+ H_2O**	^{15}N	6.8±0.08	~3.3	1.5	~2.0	15.3 ±0.1	208±6	~19, ~46	~0.2, ~0.1
**0.50 M Urea+ H_2O**	^{13}Ca	7.6±0.42	~5.0	1.4	~1.7	15.3 ±0.4	174±8	~22, ~13	~0.1, ~20

*, "~" is used to indicate that the results were estimated as p_I, k_{UI}, k_{IU}, k_{NI}, and k_{IN} are correlated in the fitting.

Table S4. Folding and unfolding parameters extracted with the three-state model U-N-I.

Solvents	Expt.	p_u (%)	p_I (%)	ΔG_{NU} (kcal/mol)	ΔG_{NI} (kcal/mol)	k_{NU} (s^{-1})	k_{UN} (s^{-1})	k_{NI} +k_{IN} (s^{-1})
**D_2O**	^{13}Ca	2.0±0.01	~2.4*	2.3	~2.2*	10.4±1.7	494.3±9.1	~23*
**H_2O**	^{13}Ca	4.4±0.02	~2.4	1.8	~2.2	16.8±0.9	366.9±10.6	~47
**H_2O**	^{15}N	4.7±0.01	~5.3	1.8	~1.7	15.6±0.3	302.4±4.3	~3
**0.25 M Urea+H_2O**	^{13}Ca	6.4±0.05	~2.4	1.6	~2.2	15.6±0.7	231.3±8.5	~35
**0.25 M Urea+H_2O**	^{15}N	6.5±0.06	~7.8	1.5	~1.4	15.2±0.3	202.4±2.9	~13
**0.50 M Urea+H_2O**	^{13}Ca	7.9±0.04	~13.3	1.4	~1.1	16.1±0.8	170.1±3.9	~14

*, "~" is used to indicate that the results were estimated as p_I and $(k_{UI} + k_{IU})$ are correlated in the fitting.

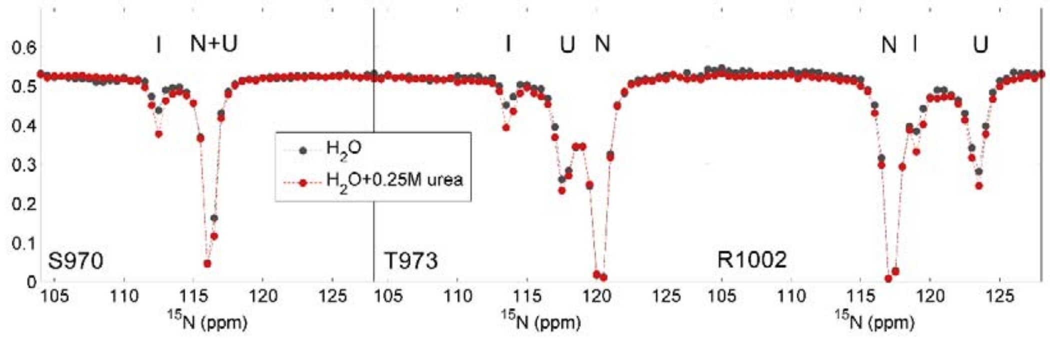

Figure S1. Comparison of ^{15}N CEST profiles in H_2O and 0.25 M urea.

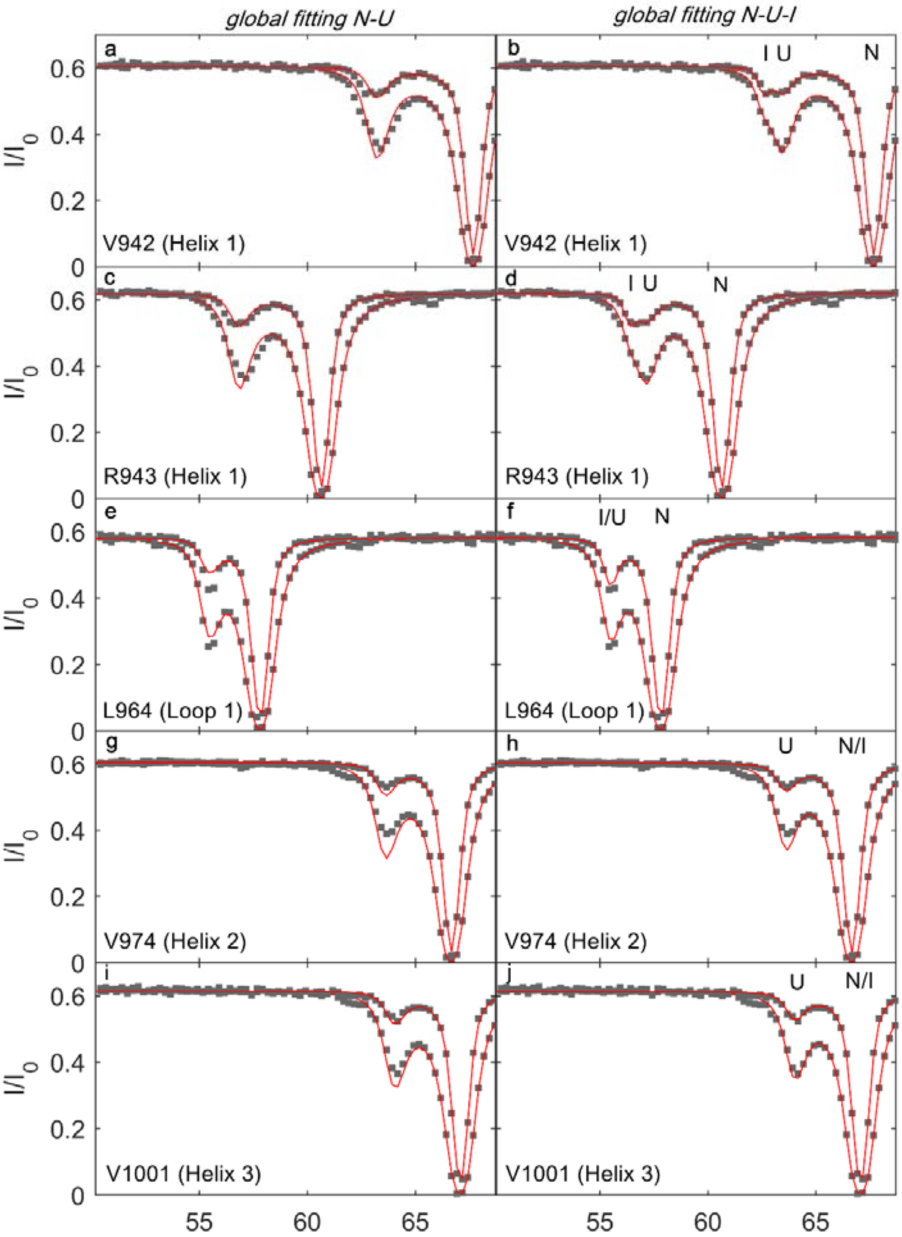

Figure S2. Examples of D2O [13]Ca CEST ***global fitting*** using the two-state model N-U (*left: a,c,e,g,i*) and three-state model N-U-I (*right: b,d,f,h,j*). Grey squares are experimental data, red lines are calculated profiles based on ***global fitting*** using the two-state model (or three-state model), where all residues share same k_{ex} and p_u (and p_I). The fitting based on the two-state model was bad, especially for the minor dip regions. By comparing profiles of residues in C- and N-terminal regions, we can see obvious differences in the depth of the minor state, which indicate the differences of p_u for the two regions.

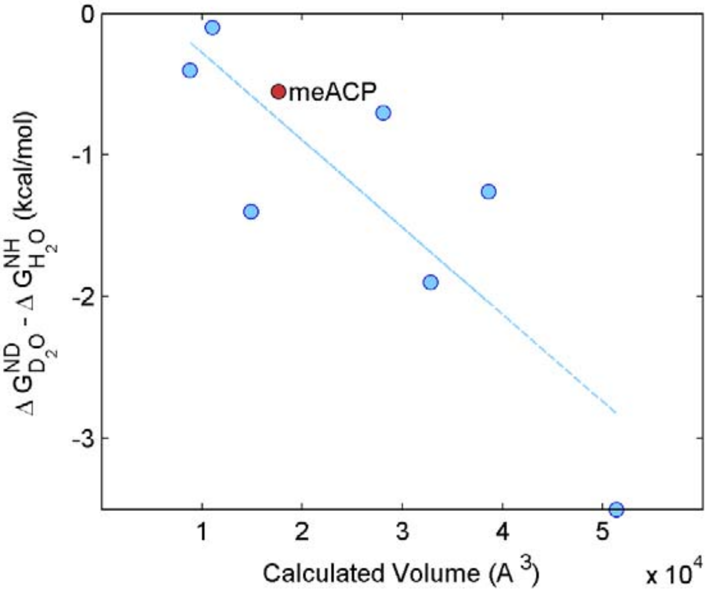

Figures S3. Correlation of H/D solvent isotope effect with protein size.

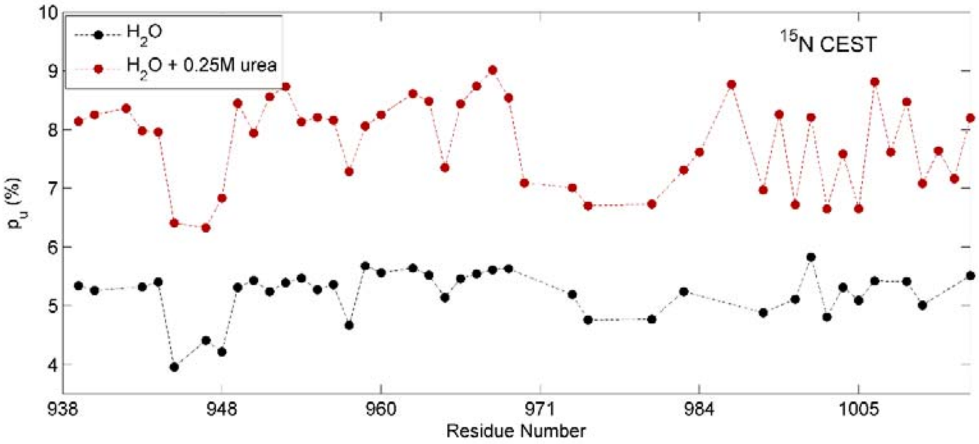

Figure S4. Populations of state U (p_u) extracted from individual ^{15}N CEST profiles recorded in H_2O and 0.25 M urea. The two-state model (M1) was used in the extraction of p_u.

References

1 Krantz, B. A. *et al.* Understanding protein hydrogen bond formation with kinetic H/D amide isotope effects. *Nat. Struct. Mol. Biol.* **9**, 458-463 (2002).

2 Parker, M. J. & Clarke, A. R. Amide backbone and water-related H/D isotope effects on the dynamics of a protein folding reaction. *Biochemistry* **36**, 5786-5794 (1997).

3 Kuhlman, B. & Raleigh, D. P. Global analysis of the thermal and chemical denaturation of the N - terminal domain of the ribosomal protein L9 in H2O and D2O. Determination of the thermodynamic parameters, ΔH°, ΔS°, and ΔC° p, and evaluation of solvent isotope effects. *Protein Sci.* **7**, 2405-2412 (1998).

4 Scheiner, S. & Cuma, M. Relative stability of hydrogen and deuterium bonds. *J. Am. Chem. Soc.* **118**, 1511-1521 (1996).

5 Lim, J. *et al.* Solution Structures of the Acyl Carrier Protein Domain from the Highly Reducing Type I Iterative Polyketide Synthase CalE8. *Plos One* **6**, e20549 (2011).

6 Krantz, B. A., Moran, L. B., Kentsis, A. & Sosnick, T. R. D/H amide kinetic isotope effects reveal when hydrogen bonds form during protein folding. *Nat. Struct. Mol. Biol.* **7**, 62-71 (2000).

7 Huyghues-Despointes, B. M., Scholtz, J. M. & Pace, C. N. Protein conformational stabilities can be determined from hydrogen exchange rates. *Nat. Struct. Mol. Biol.* **6**, 910-912 (1999).

8 Sato, S. & Raleigh, D. P. Kinetic isotope effects reveal the presence of significant secondary structure in the transition state for the folding of the N-terminal domain of L9. *J. Mol. Biol.* **370**, 349-355 (2007).

9 Efimova, Y., Haemers, S., Wierczinski, B., Norde, W. & Van Well, A. Stability of globular proteins in H2O and D2O. *Biopolymers* **85**, 264-273 (2007).
